# Serum IGF-1 is associated with cognitive functions in early, drug-naïve Parkinson’s disease

**DOI:** 10.1371/journal.pone.0186508

**Published:** 2017-10-24

**Authors:** Marina Picillo, Rosario Pivonello, Gabriella Santangelo, Claudia Pivonello, Riccardo Savastano, Renata Auriemma, Marianna Amboni, Sara Scannapieco, Angela Pierro, Annamaria Colao, Paolo Barone, Maria Teresa Pellecchia

**Affiliations:** 1 Center for Neurodegenerative Diseases (CEMAND), Department of Medicine and Surgery, Neuroscience Section, University of Salerno, Salerno, Italy; 2 Department of Clinical Medicine and Surgery, Federico II University, Naples, Italy; 3 Neuropsychology Laboratory, Department of Psychology, Second University of Naples, Caserta, Italy; 4 IOS and Coleman Medicina Futura Medical Center, Naples, Italy; 5 IDC Hermitage-Capodimonte, Naples, Italy; Philadelphia VA Medical Center, UNITED STATES

## Abstract

**Objective:**

Cognitive deficits are common in Parkinson’s disease (PD) since the early stages and many patients eventually develop dementia. Yet, occurrence of dementia in PD is unpredictable. Evidence supports the hypothesis that insulin-like growth factor-1 (IGF-1) is involved in cognitive deficits. Our aim was to evaluate the relationship between serum IGF-1 levels and neuropsychological scores in a large cohort of drug-naïve PD patients during the earliest stages of the disease.

**Methods:**

Serum IGF-1 levels were determined in 405 early, drug-naïve PD patients and 191 healthy controls (HC) enrolled in the Parkinson’s Progression Markers Initiative (PPMI). The association between serum IGF-1 levels and neuropsychological scores was evaluated with linear regression analysis.

**Results:**

IGF-1 levels were similar in PD and HC. In PD patients the lowest IGF-1 quartile was a predictor of lower performances at the Semantic Fluency task (β = -3.46, 95%CI: -5.87 to -1.01, p = 0.005), the Symbol Digit Modalities Score (β = -2.09, 95%CI: -4.02 to -0.15, p = 0.034), and Hopkins Verbal Learning Test Retention (β = -0.05, 95%CI: -0.09 to -0.009, p = 0.019).

**Conclusions:**

Lower serum IGF-1 levels are associated to poor performances in cognitive tasks assessing executive function, attention and verbal memory in a large cohort of early PD patients. Follow-up studies are warranted to assess if IGF-1 is related to the development of dementia in PD.

## Introduction

Cognitive disturbances are common findings in Parkinson’s disease (PD) since the earliest stages, manifesting with executive as well as with visuospatial deficits [1]. Cognitive function typically decline over time, with many patients eventually developing dementia [2]. Evolution to dementia is a key milestone in PD progression as denotes a subgroup of patients with worse prognosis and represents a heavy burden for both patients and caregivers. Among all, age, male gender and presence of subtle cognitive impairment have been identified as the most consistent predictors of dementia [2]. Despite considerable efforts of the research community, valid biomarkers of cognitive impairment for PD have yet to come. As minimally invasive and economically affordable, serum biomarkers for neurodegenerative diseases gained increasing attention in recent years.

Both preclinical and clinical evidence support the hypothesis that Insulin-like growth factor 1 (IGF-1) is involved in neuroprotection and plays a role in determining cognitive deficits: a) animal and in vitro studies show that IGF-1 is critical for neuronal cell functioning, enhancing neuronal survival and inhibiting apoptosis and B-amyloid deposition [3–5]; b) studies disclose cognitive impairment in children with growth hormone (GH) deficit [6]; c) cross-sectional epidemiologic studies report an association between low IGF-1 levels and reduced cognitive performance in healthy, elderly subjects [7,8], with particular regard to executive function, working memory and attention tasks [9]; d) prospective studies demonstrate that higher serum IGF-1 levels are associated with less cognitive decline in the elderly [10–13].

In keeping with this evidence, we reported a significant association between low serum IGF-1 levels and poor performances on executive tasks at PD diagnosis and on attention/executive and verbal memory tasks after two-years since diagnosis in a cohort of 65 drug-naïve, early patients, suggesting a link between IGF-1 and early cognitive impairment in PD [14].

In the present study, we sought to analyze the relationship between serum IGF-1 levels and cognitive scores at diagnosis in a larger cohort of drug-naïve PD patients. Based on previous evidence, lower serum IGF-1 levels were expected to be associated with worse cognitive performances. Furthermore, the association between IGF-1 levels and cognition was explored in a large cohort of healthy controls (HC).

## Methods

Data used for this study were obtained from the Parkinson’s Progression Markers Initiative (PPMI) database (http://www.ppmi-info.org/data). PPMI-a public-private partnership-is funded by the Michael J. Fox Foundation for Parkinson’s Research and funding partners (www.ppmi-info.org/fundingpartners). The aims and methodology of the study are available at www.ppmi-info.org/study-design. The study was approved by the institutional review board at each site, and participants provided written informed consent. For up-to-date information on the study visit www.ppmi-info.org.

PD patients were enrolled in the PPMI study provided they were drug-naïve, had less than 2-years disease duration and evidence of dopamine transporter (DAT) deficit on DAT Scan imaging. Enrolled subjects underwent scheduled assessments to collect clinical data and biospecimens every 3 months for the first year and then every 6 months up to 5 years. Disease severity has been assessed by means of the Movement Disorders Society version of the Unified Parkinson’s disease rating scale part III (MDS-UPDRS-III).

Baseline serum samples for the IGF-1 analysis were obtained from the PPMI (Cognition biomarkers, 2013; Grant ID 8800; http://www.ppmi-info.org/access-data-specimens/request-specimens/). Demographic, clinical and neuropsychological data were gathered from the PPMI database according to guidelines for data access and use (http://www.ppmi-info.org/data, S1 File).

According to PPMI Laboratory Manual, venous blood samples from PD patients and HC were drawn at baseline visit in the morning after an overnight fast. Blood samples were centrifuged and serum was frozen (-80°C). Serum samples from 405 PD patients and 191 HC collected at the baseline assessment of the PPMI were shipped on dry ice and analyzed at the Department of Clinical Medicine and Surgery, Federico II University, Naples.

### IGF-1 measurement

We performed the IGF-1 measurement using the Quantikine Human IGF-1 Immunoassay (R&D Systems, Minneapolis, MN, USA), that is a solid-phase ELISA designed to measure human IGF-1 in serum and plasma. The immunoassay is calibrated against a highly purified Escherichia coli-expressed recombinant human IGF-1. To reduce analytical variance, samples were batch analyzed using the same assay lot. Serum was pretreated to release IGF-1 from IGF-binding proteins with acid-ethanol extraction. Each sample was tested twice in the same assay and the mean of these two values was considered for statistical analysis. The NIBSC/WHO IGF-1 International Reference Reagent 02/254 was evaluated in this assay. The minimal detectable dose (MDD) of IGF-1 in this assay ranged from 0.007 to 0.056 ng/ml and the mean MDD was 0.026 ng/ml. The intra-assay coefficients of variation (CVs) were 3.5%, 4.3% and 4.3%, and the inter-assay CVs were 8.1%, 8.3% and 7.5% for low, medium and high points of the standard curve, respectively.

### Cognitive evaluation

Global cognition was screened with the Montreal Cognitive Assessment (MoCA) [15]. An extensive battery of neuropsychological tests was administered to evaluate different cognitive domains. In detail, memory was tested with the Hopkins Verbal Learning Test-Revised (HVLT-R) [16]; visuospatial function with the Benton Judgment of Line Orientation (JOLO) 15-item version [17]; processing speed-attention with the Symbol-Digit Modalities Test (SDMT) [18]; and executive function and working memory with Letter-Number Sequencing (LNS) [19] and semantic fluency (SF) [20]. With regard to HVLT-R, Immediate Recall (IR), Delayed Recall (DLRY), Retention and Discrimination recognition (DR) were analyzed. Language domain was not assessed.

### Statistical analysis

Differences in the distribution of categorical variables among groups were assessed by the chi-square test. After Kolmogorov-Smirnov testing for normal distribution, parametric testing was performed for group comparisons (independent sample t-test or one-way ANOVA with post-hoc Bonferroni test, as applicable). Descriptive statistics are given as means and standard deviation (SD) with range.

Those variables of interest assessing specific cognitive domains (ie, all except the MoCA score) reaching a significance threshold≤0.1 further underwent linear regression analysis to evaluate the association between serum IGF-1 levels and cognitive scores. Based on the observation that only the lowest quartile of IGF-1 tended to present worse cognitive outcomes compared to the other three quartiles (and Table 1), further analysis were performed comparing the lowest IGF-1 quartile to all other quartiles combined. Visual inspection of graphs also confirmed the lowest IGF-1 quartile tended to present worse scores. Linear regression analysis included age, education, gender as covariates with no interaction terms. Education years were divided into 2 groups based on the median (16 years); group 0: ≤16 years, group 1: >16 years. Results were considered statistically significant at *p*<0.05.

**Table 1:** Characterization of the PD cohort according to IGF-1 quartiles.

		Total cohort (405)	The lowest quartile (110)	The second quartile (86)	The third quartile (100)	The highest quartile (109)	p
**IGF-1, ng/ml**		33.800–412.200	≤ 97.9	97.9001–124	124.0001–162.200	≥ 162.2001	NA
**Demographic and motor variables**							
**Age, years**	61.20 (9.76) (33, 84)		63.33 (9.04) (33, 85)	60.62 (11.63) (33, 82)	62.14 (11.91) (33, 83)	61.07 (10.44) (33, 82)	0.278
**Gender (M/F)**	264/141		58/52 (52.70/47.30)	62/24 (72.10/27.90)	65/35 (65/35)	79/30 (72.50/27.50)	<0.05*
**BMI**	27.20 (5.10) (16.80, 45)		26.92 (6.62) (18.18, 45)	28.01 (4.64) (19.65, 41.57)	27.21 (4.61) (17.72,41.05)	26.95 (3.91) (16.80, 36.85)	0.437
**Education, years**	15.56 (2.98) (5, 26)		15.25 (3.35) (5, 22)	15.84 (3.37) (8, 26)	15.39 (2.67) (8, 24)	15.76 (2.53) (11, 23)	0.443
**Disease duration, months**	6.33 (6) (0, 35)		5.8 (4.31) (0, 28)	5.92 (5.71) (4, 35)	6.21 (5.81) (6, 30)	6.51 (5.72) (8, 28)	0.499
**MDS-UPDRS-III**	20.25 (8.93) (4, 51)		21.03 (9.38) (6, 51)	20.87 (7.42) (6, 39)	18.44 (8.52) (6, 45)	20.55 (9.81) (4, 49)	0.140
**Cognitive variables**							
**MoCA, total score**	27.10 (2.34) (17, 30)		27 (2.37) (19, 30)	27.02 (2.32) (17, 30)	27.62 (2.08) (21, 30)	26.86 (2.51) (17, 30)	0.078
**JOLO**	12.69 (2.23) (5, 15)		12.81 (2.41) (5,15)	12.76 (2.28) (5,15)	12.96 (2.08) (7,15)	12.3 (2.13) (6, 15)	0.164
**HVLT IR**	24.45 (4.99) (9, 36)		24.11 (4.88) (11, 32)	24.52 (5.15) (14, 34)	25.49 (4.89) (9, 35)	24.86 (5.02) (13, 36)	*0*.*097*^§^
**HVLT DRLY**	8.36 (2.52) (0, 12)		7.95 (2.72) (0, 12)	8.56 (2.14) (3, 12)	8.79 (2.57) (0, 12)	8.22 (2.51) (0, 12)	*0*.*084*^
**HVLT retention**	0.85 (0.20) (0, 1.29)		0.81 (0.22) (0, 1.22)	0.89 (0.16) (0.44, 1.29)	0.86 (0.19) (0, 1.22)	0.85 (0.21) (0, 1.29)	*0*.*074*°
**HVLT DR**	9.65 (2.54) (-2, 12)		9.51 (2.52) (-1, 12)	9.73 (2.52) (-1, 12)	9.89 (2.54) (0, 12)	9.55 (2.61) (-2, 12)	0.683
**LNS**	10.56 (2.66) (2–20)		10.15 (2.92) (4, 20)	10.41 (2.56) (2, 16)	10.68 (2.71) (3, 20)	10.98 (2.38) (5, 17)	0.120
**Semantic fluency**	48.52 (11.72) (20, 103)		46.31 (10.73) (20, 75)	49.15 (12.19) (26, 83)	50.13 (11.37) (25, 91)	48.77 (12.36) (24, 103)	*0*.*100*^$^
**SDMT**	41.17 (9.78) (7, 82)		38.89 (9.88) (7, 61)	41.76 (10.74) (20, 82)	41.77 (8.9) (16, 70)	42.54 (9.35) (17, 66)	*0*.*036*^&^

Data are in mean (SD) (range), unless otherwise specified. Significance level≤0.1 are shown in italics.

* p = 0.005 for the lowest versus the second quartile; p = 0.002 for the lowest versus the fourth quartile.

^§^ -0.403, p = 0.937 for the lowest versus the second quartile; -1.370, p = 0.286 for the lowest versus the third quartile; -0.256, p = 0.989 for the lowest versus the highest quartile.

^ -0.615, p = 0.543 for the lowest versus the second quartile; -0.835, p = 0.101 for the lowest versus the third quartile; -0.266, p = 0.787 for the lowest versus the highest quartile.

° -0.075, p = 0.060 for the lowest versus the second quartile; -0.045, p = 0.608 for the lowest versus the third quartile; -0.040, p = 0.811 for the lowest versus the highest quartile

^$^ -2.848, p = 0.547 for the lowest versus the second quartile; -3.827, p = 0.109 for the lowest versus the third quartile; -2.468, p = 0.714 for the lowest versus the highest quartile

^&^ -2.866, p = 0.247 for the lowest versus the second quartile; -2.88, p = 0.196 for the lowest versus the third quartile; -3.560, p = 0.042 for the lowest versus the highest quartile

BMI: Body Mass Index; HVLT DLRY: Hopkins Verbal Learning Test Delayed Recall; HVLT DR: Hopkins Verbal Learning Test Discrimination Recognition; HVLT IR: Hopkins Verbal Learning Test Immediate Recall; JOLO: Benton Judgment of Line Orientation (15-item version); LNS: Letter Number Sequencing; MDS-UPDRS-III: Movement Disorders Society version of the Unified Parkinson’s disease rating scale part III; MoCA: Montreal Cognitive Assessment; SDMT: Symbol Digit Modalities Test.

## Results

Demographic and clinical data of PD patients are reported in Table 1. Serum IGF-1 and age were not different between PD and HC (136.6±56.1 vs 134.45±56.13 ng/ml; p = 0.64). Serum IGF-1 levels were divided in quartiles as follows: lowest quartile ≤ 97.9 ng/ml; second quartile = 97.9001–124 ng/ml; third quartile = 124.0001–162.200 ng/ml; highest quartile ≥ 162.2001 ng/ml.

**Fig 1:**
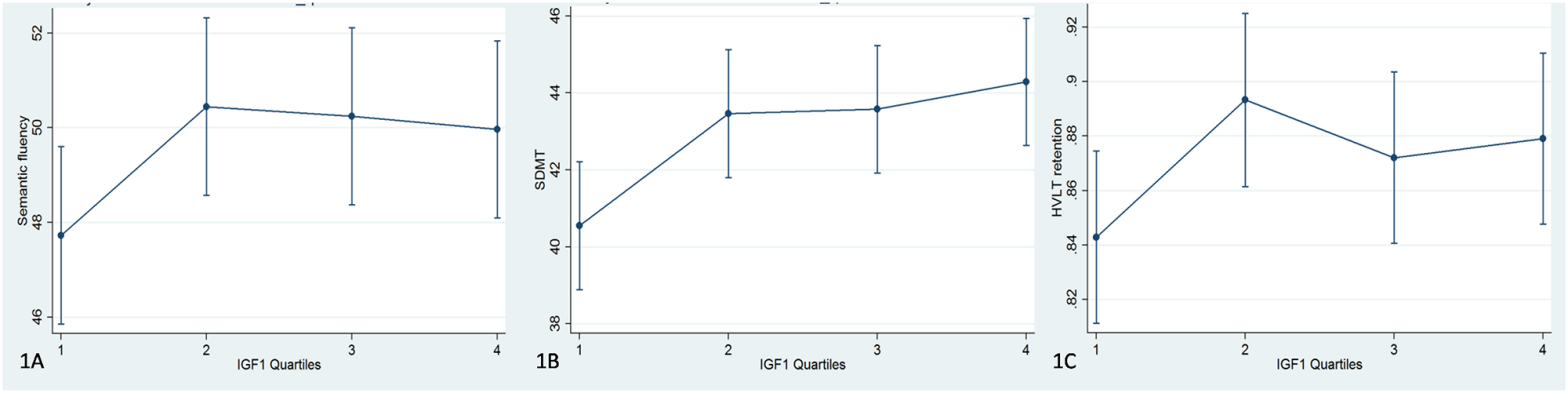
Box plots showing differences in cognitive performances in different IGF-1 quartiles (1A = Semantic fluency; 1B = Symbol-Digit Modalities Test; 1C = Hopkins Verbal Learning Test-Retention). Yaxis shows cognitive scores adjusted for age, gender and education according to the regression analysis. According to one-way ANOVA, the lowest quartile presents worse performances as compared to the other quartiles. *: p<0.001. Outliers are showed as circles.

Linear regression analysis showed that in PD patients the lowest IGF-1 quartile was associated to worse performances in several cognitive tasks compared to all the others quartiles (Table 2). In detail, the lowest IGF-1 quartile was a predictor of lower scores at the SF task (p = 0.005), along with age (p<0.001), male gender (p<0.001) and education (p = 0.001) (Fig 1A). Lower performances at the SDMT were associated to the lowest IGF-1 quartile (p = 0.034), age (p<0.001) and male gender (p = 0.001) (Fig 1B). Finally, the lowest IGF-1 quartile was a predictor of lower performances at the HVLT Retention (p = 0.019), along with age (p = 0.001) and male gender (p = 0.003) (Fig 1C).

**Table 2:** Significant predictors of cognitive performances in PD patients at baseline.

	Parameter estimate (95% CI)	p
***Semantic Fluency***		
IGF-1 lowest quartile	-3.46 (-5.87, -1.01)	0.005
Gender	-7.08 (-9.31, -4.84)	<0.001
Age	-0.29 (-0.40, -0.18)	<0.001
Education	3.85 (1.61, 6.09)	0.001
***Symbol Digit ModalitiesTest***		
IGF-1 lowest quartile	-2.09 (-4.02, -0.15)	0.034
Age	-0.44 (-0.53,-0.36)	<0.001
Gender	-3.03 (-4.81, -1.24)	0.001
Education	0.92 (-0.87, 2.71)	0.313
***HVLT retention***		
IGF-1 lowest quartile	-0.05 (-0.09,-0.009)	0.019
Age	-0.004 (-0.006, -0.001)	0.001
Gender	-0.06 (-0.10, -0.02)	0.003
Education	0.04 (0.00, 0.08)	0.052

CI: confidence interval; HVLT: Hopkins Verbal Learning Tes

No significant associations were found between IGF-1 and any of the neuropsychological tasks in HC.

## Discussion

Confirming our hypothesis, we demonstrated that lower serum IGF-1 levels are associated with lower performances on cognitive tasks in a large cohort of early, drug-naïve PD patients. In detail, lower serum IGF-1 levels are linked to poor performances in cognitive tasks assessing executive function, attention and verbal memory. Deficits in such tasks are driven by a dysfunction in the prefrontal and temporal cortex. In detail, neuroimaging studies suggest that while the capability of holding information and sustaining attention (evaluated with the retention and delayed recall of words list and the SDMT) are both ensured by the prefrontal cortex [21], the ability to access the conceptual knowledge stores (evaluated by the Semantic fluency task) is provided by the temporal cortex [22]. Therefore, our data suggest that low IGF-1 levels are linked to impaired cognitive functions (i.e. cognitive flexibility, attention, devising a search strategy) mediated by prefrontal and temporal cortex.

In spite of using different neuropsychological tests, these results are consistent with our previous study in a cohort of 65 drug-naïve, early PD patients showing a significant correlation between low serum IGF-I levels and poor performance on both executive tasks at baseline and attention/executive and verbal memory tasks at 2-year follow-up [14]. Therefore, taken together the findings from the current and previous study support the link between IGF-1 and cognitive impairment in PD patients even in the earliest stages of disease.

Although explored in a spoonful of studies [14,23], the relation between IGF-1 and cognitive performance in PD is not surprising. Indeed, a relevant number of IGF-1 receptors have been reported in several brain areas essential for cognitive performance, with highest concentrations in the hippocampus and the frontal cortex [3,24,25]. Preclinical evidence also suggest, IGF-1 plays out an active defence mechanism against degeneration of the brain [26]. As a matter of fact, executive functioning, memory, attention and verbal fluency present specific improvement in GH-deficient populations treated with hormone replacement therapy [27].

Several preclinical and clinical studies highlighted a link between IGF-1 and neurodegeneration and demonstrated that it may play an important neuroprotective role by various mechanisms [28]. Indeed, animal and in vitro studies showed that IGF-1 enhances neuronal survival and inhibits apoptosis. In both Alzheimer’s disease (AD) models and patients, IGF-1 has been suggested to increase Β-amyloid clearance and protect neurons against Β-amyloid toxicity [4,5]. Interestingly, a recent study including more than three thousands subjects from the Framingham community showed that healthy subjects with IGF-1 in the lowest quartile had a 51% greater risk of developing AD and, among persons without dementia, higher levels of IGF-1 were associated with greater brain volumes, strongly suggesting that higher levels of IGF-1 may protect against subclinical and clinical neurodegeneration [29]. As regards relationship between IGF-1 and α-synuclein pathology, IGF-1 can rescue α-synuclein toxicity and suppress α-synuclein aggregation through the activation of Akt pathway in cultured cells [30,31].

Indeed, the pathophysiology of cognitive impairment in PD is complex. It involves multiple neurotransmitter systems and diffuse neurodegeneration, and significant gaps still remain in our knowledge of cognitively impaired, non-demented PD patients [32,33]. We acknowledge the range of neuropsychological findings in patients attributed to different IGF-1 quartiles is remarkable with a great overlap. Although our analysis took into account several additional factors possibly influencing cognitive performances in PD patients as age, gender and education, still several aspects were omitted. However, as there is a dearth of consistent serum biomarkers in PD, our intent was to focus on the role of serum IGF-1 as possible marker of cognitive impairment in a large cohort of early stage PD. Future studies should clarify the strength of the relationship between IGF-1 and cognitive performances when considering other clinical, genetic and neuroimaging markers.

In contrast with previous findings [34,35], we found IGF-1 levels similar in PD and HC, implying this is not a valid biomarker for PD diagnosis. Godau et al first detected higher IGF-1 levels in PD compared to healthy subjects and proposed IGF-1 as a marker for PD diagnosis [36,37]. Then, we replicated their findings in our small cohort of de novo PD patients [14,38]. On the other hand, Numao et al already reported no difference when comparing IGF-1 levels between Japanese PD patients and healthy subjects [39]. Indeed, the present study is the larger conducted so far and the sample size may in part account for the discrepancy with previous evidence.

Several confounding factors may affect IGF-1 levels including body mass index (BMI), diabetes, cancer, thyroid dysfunction, inflammatory diseases and medications as corticosteroids [14,35,38]. We identified such subjects in the PD PPMI cohort (diabetes: 17; cancer: 11; thyroid dysfunction: 59; on corticosteroids: 4) and repeated all the analysis after their exclusion. However, results were largely confirmed. In addition, such subjects did not show different IGF-1 values. Finally, the different IGF-1 quartiles did not display significant differences in terms of BMI (Table 1).

Our study has some limitations. First, our analysis focused only on the baseline assessments of the PPMI study and, thus, included patients with relatively preserved cognitive performances. Being the cognitive performances of recruited patients relatively stable over the first 2-year follow up, it is challenging nowadays to establish the real significance of any biomarker of cognitive impairment. However, the PPMI cohort is currently being followed and the next step is to verify if baseline biomarkers levels can predict the onset of PD with dementia on the long-term follow up. Second, in spite of being the larger existing cohort of de novo PD patients, subjects enrolled in the PPMI study may not completely represent the respective counterparts in standard clinical settings. Differently from a previous study analyzing preliminary data from the PPMI cohort (63 PD and 39 HC), no major differences in cognitive performances have been observed between PD and HC [40,41]. It should be noted that the PPMI PD cohort comprises predominantly high-educated volunteers committed to clinical examinations, neuroimaging and longitudinal follow-ups. As such, PD patients are generally more educated and present better cognitive performances as compared to previous, smaller cohorts of PD patients. Thus, our results may be not unhesitatingly generalizable to all PD patients. On the other hand, PPMI database ensures high data quality collection. Finally, future studies evaluating the impact of a combined set of biomarkers (eg, IGF-1 and ApoE4 carrier status) are warranted.

In conclusion, this is the first large study to indicate a relationship between IGF-1 and specific cognitive functions in early PD patients. Long-term follow up of the same cohort will clarify if serum biomarkers represent useful tools for the early detection of dementia in PD.

## Supporting information

S1 File Recruitment category: 0 = healthy controls, 1 = Parkinson’s disease; IGF_1 = IGF-1 values; gender: 0 = women, 1 = men; date of birth = year of birth; education = years of education; MDS_UPDRS_part_I_TOT: Movement disorders society_unified Parkinson’s disease part I; MDS_UPDRS_part_II_TOT: Movement disorders society_unified Parkinson’s disease part II; MDS_UPDRS_part_III_TOT: Movement disorders society_unified Parkinson’s disease part III; MDS_UPDRS_Total score: Movement disorders society_unified Parkinson’s disease total score; age at assessment = age at assessment; MOCATOT: Montreal Cognitive Assessment battery total score; Benton: Benton Judgment of Line Orientation; HVLT_immediate_recall: Hopkins Verbal Learning Test-Revised immediate recall; HVLT-R DR: Hopkins Verbal Learning Test-Revised discrimination recognition; LNS: Letter-Number Sequencing; SF: Semantic fluency; SDMT: Symbol-Digit Modalities Test; HVLT-R DLRY: Hopkins Verbal Learning Test-Revised delayed recall.
(XLS)

## Abbreviations

HVLT-R DLRY: Hopkins Verbal Learning Test-Revised delayed recall
HVLT-R DR: Hopkins Verbal Learning Test-Revised discrimination recognition
HVLT-R IR: Hopkins Verbal Learning Test-Revised immediate recall
HVLT-R: Hopkins Verbal Learning Test-Revised
IGF-1: insulin-like growth factor-1
JOLO: Benton Judgment of Line Orientation
LNS: Letter-Number Sequencing
MDS-UPDRS-III: Movement Disorders Society version of the Unified Parkinson’s disease rating scale part III
MoCA: Montreal Cognitive Assessment
PD: Parkinson’s disease
PPMI: Parkinson’s Progression Markers Initiative
SD: standard deviation
SDMT: Symbol-Digit Modalities Test
SF: Semantic fluency
